# Clinical and socioeconomic determinants of glycaemic control derived from continuous glucose monitoring in adults with type 1 diabetes

**DOI:** 10.3389/fendo.2026.1813510

**Published:** 2026-04-23

**Authors:** Ángel Manuel Mesa Díaz, Pablo Rodríguez de Vera Gómez, María Ruiz Rodríguez, Samuel Belmonte Lomas, Blanca Gómez Zaragoza, Eduardo Mayoral Sánchez, María Asunción Martínez Brocca

**Affiliations:** 1 Department of Endocrinology and Nutrition, Virgen Macarena University Hospital, Seville, Spain; 2 Andalusian Diabetes Plan, Ministry of Health and Consumer Affairs, Regional Government of Andalusia, Seville, Spain

**Keywords:** ambulatory glucose profile, continuous glucose monitoring, glycaemic variability, socioeconomic status, time in range, type 1 diabetes

## Abstract

**Aim:**

To identify clinical, sociodemographic and glucometric factors associated with achieving international glycaemic targets derived from intermittently scanned continuous glucose monitoring (isCGM) in adults with type 1 diabetes mellitus (T1DM) treated with multiple daily injections (MDI) in Andalusia, a region with universal access to isCGM.

**Methods:**

A cross-sectional, population-based study was conducted using centralized electronic health records from the Andalusian Public Health System (APHS). Adults with T1DM using isCGM for ≥1 year and integrated glucometric data in Electronic Health Records in APHS in the 14 days preceding the download were included. Four glycaemic endpoints were evaluated: time in range (TIR ≥ 70%), time above range (TAR < 25%), time below range (TBR < 5%), and achievement of full AGP profile. Univariate analyses and multivariable logistic regression models were used to identify independent predictors.

**Results:**

A total of 7,885 individuals were included. Overall, 24.8% achieved TIR ≥ 70%, 35.0% TAR < 25%, 72.9% TBR < 5%, and 12.3% met the full AGP profile. Lower HbA_1c_, lower glycaemic variability and a higher number of daily scans were consistently associated with achieving glycaemic targets. Older age and male sex were independent predictors of TIR ≥ 70%. Socioeconomic status showed a significant association: individuals in the intermediate-income group had higher odds of achieving TIR, TAR and full AGP criteria. No socioeconomic associations were observed for TBR < 5%.

**Conclusions:**

In a real-world setting with universal isCGM provision, clinical, sociodemographic and glucometric determinants independently influence the achievement of glycaemic targets in adults with T1DM. Integrating advanced monitoring metrics with explicit consideration of social determinants may enhance the precision and equity of glycaemic management strategies.

## 1 Introduction

Achieving optimal glycaemic control within target ranges in type 1 diabetes (T1D) is a key determinant in reducing the incidence of both acute and chronic complications, as well as in improving patients’ quality of life (1). Although glycated haemoglobin (HbA_1c_) has become the standard marker of metabolic control, its ability to capture glycaemic variability and the frequency of hypo- and hyperglycaemic episodes is limited (2).

The development and widespread adoption of intermittently scanned continuous glucose monitoring (isCGM) systems have transformed the clinical approach to glycaemic management, enabling a dynamic and individualized assessment of patients’ glucose profiles (3, 4). In this context, the standardized use of the ambulatory glucose profile (AGP) has been instrumental, providing both graphical and quantitative representations of daily glucose distributions and facilitating the clinical interpretation of monitoring-derived data. These technologies have driven the adoption of key metrics such as time in range (TIR), time above range (TAR), time below range (TBR), and coefficient of variation (CV), which have been endorsed by international consensus statements as priority targets for glycaemic control due to their close association with microvascular complication risk and multiple quality-of-life indicators (5, 6).

Although clinical and behavioural determinants of glycaemic control have been extensively described in terms of HbA_1c_ (7), their impact on these newer glucose monitoring metrics remains insufficiently explored. Moreover, among these predictors, the influence of sociodemographic and socioeconomic characteristics continues to be poorly characterised (8). In this regard, income level constitutes a particularly relevant marker (9).

In Andalusia, a region in southern Spain with 8.4 million inhabitants (approximately 18% of the national population), the Andalusian Public Health System (APHS) provides universal coverage for people with diabetes treated with multiple daily insulin injections (MDI) through a standardized implementation programme previously described by our group (10–12). This homogeneous, large-scale deployment offers a unique opportunity to evaluate the real-world clinical outcomes of isCGM use (12, 13).

The aim of the present study is to identify the clinical, sociodemographic, and glucometric factors associated with achieving internationally agreed glycaemic control targets in adults with T1D treated with MDI and using isCGM in Andalusia, through a population-based cross-sectional study.

## 2 Materials and methods

### 2.1 Study design, variables, and data sources

A population-based, observational cross-sectional study was conducted in Andalusia, including adults with T1D treated with MDI and using isCGM. Eligible participants were adults aged ≥ 18 years with a diagnosis of T1D according to the criteria of the American Diabetes Association (14), with a diabetes duration of at least one year and receiving MDI therapy. To ensure the quality of glycaemic data, continuous use of isCGM for ≥ 1 year was required, along with a minimum data capture of 70% during the 14 days preceding the analysed download. Individuals under 18 years of age, pregnant women, and patients treated with continuous subcutaneous insulin infusion (CSII), regardless of the level of automation, were excluded.

The study population was identified from all individuals with T1D who had at least one glucometric data download from the FreeStyle Libre 2 isCGM system integrated into the electronic health record during the study period. Data collection was performed retrospectively using integrated sources from the electronic health record of the APHS, with a data cut-off date of 15 May 2022. Identification of patients with T1D using isCGM was based on the corporate registry created by the APHS in 2020 for the management of large-scale implementation of this technology. Basic sociodemographic and clinical variables (age, sex, age at diagnosis, diabetes duration, and estimated income level) were obtained from the APHS User Database and Population Health Database. Income level was assessed using the pharmaceutical co-payment bracket of the Spanish National Health System as a proxy variable, which defines three income-based categories according to registered annual income: < €18,000/year, €18,000–€100,000/year, and > €100,000/year (see **Supplementary Material**). HbA_1c_ values were extracted from the Laboratory Test Module of the SSPA. All data were integrated and managed within the corporate electronic health record system of the APHS.

Clinical and sociodemographic variables collected included age, sex, age at diabetes diagnosis, diabetes duration, HbA_1c_, and annual income level.

isCGM-derived metrics included those available in the 14-day AGP reports preceding the analysed download: time in range (TIR; 70–180 mg/dL), time above range (TAR), subdivided into TAR I (181–249 mg/dL) and TAR II (≥250 mg/dL), time below range (TBR), subdivided into TBR I (55–69 mg/dL) and TBR II (≤ 54 mg/dL), mean glucose, standard deviation (SD), coefficient of variation (CV), and the Glucose Management Indicator (GMI). Based on these metrics, four final binary outcomes defining glycaemic control within target were established (1): TIR within target (> 70%) (2), total TAR (TAR I + TAR II) < 25% (3), total TBR (TBR I + TBR II) < 4%, and (4) complete AGP within target. These definitions were based on the recommendations of the international consensus on glucose monitoring metrics (Battelino et al., 2019) (5) (**Table 1**). All variables were analysed as dichotomous outcomes, classifying patients as “within target” or “out of target” according to the established thresholds.

**Table 1:** Cut-off values based on the International Consensus on Continuous Glucose Monitoring Metrics (Battelino et al., 2019).

Dependent variable	Definition	Cut-off for target achievement
Complete AGP	Simultaneous achievement of TIR, TAR, TBR	TIR >70%, TAR <25%, TBR <4%
TIR	Time in range 70–180 mg/dL	>70%
TAR	Time above range (TAR I + TAR II)	<25%
TBR	Time below range (TBR I + TBR II)	<4%

AGP, Ambulatory Glucose Profile; CV, coefficient of variation; TAR, Time Above Range; TAR I, time above range level 1 (181–250 mg/dL); TAR II, time above range level 2 (>250 mg/dL); TBR, Time Below Range; TBR I, time below range level 1 (54–69 mg/dL); TBR II, time below range level 2 (<54 mg/dL); TIR, Time in Range (70–180 mg/dL).

### 2.2 Statistical analysis

Continuous variables were described as mean and standard deviation (SD) or median and interquartile range (IQR), according to their distribution, which was assessed using the Shapiro–Wilk test. Categorical variables were expressed as absolute frequencies and percentages.

For univariate analyses examining associations between independent variables and each glycaemic control outcome (TIR, TAR, TBR, and complete AGP within target), the chi-square test or Fisher’s exact test was used for categorical variables, and Student’s t test or the Mann–Whitney U test for quantitative variables, as appropriate.

Subsequently, multivariable logistic regression models were developed for each of the four outcome variables. All models included the following covariates, selected based on their clinical and epidemiological relevance: age, sex, age at diagnosis, duration of T1D, HbA_1c_, glycaemic variability, number of daily sensor scans, and estimated income level. Results were expressed as odds ratios (ORs) with their corresponding 95% confidence intervals (95% CIs). The discriminative ability of each model was assessed using the area under the receiver operating characteristic curve (AUC).

A two-sided level of statistical significance of p < 0.05 was assumed. All analyses were performed using IBM SPSS Statistics.

### 2.3 Ethical considerations

The study was approved by the Research Ethics Committee of the Andalusian Biomedical Research Ethics Coordination Committee (202599902571973) and was conducted in accordance with the ethical principles outlined in the Declaration of Helsinki and current legislation on personal data protection.

## 3 Results

### 3.1 General characteristics of the study population

**Figure 1:**
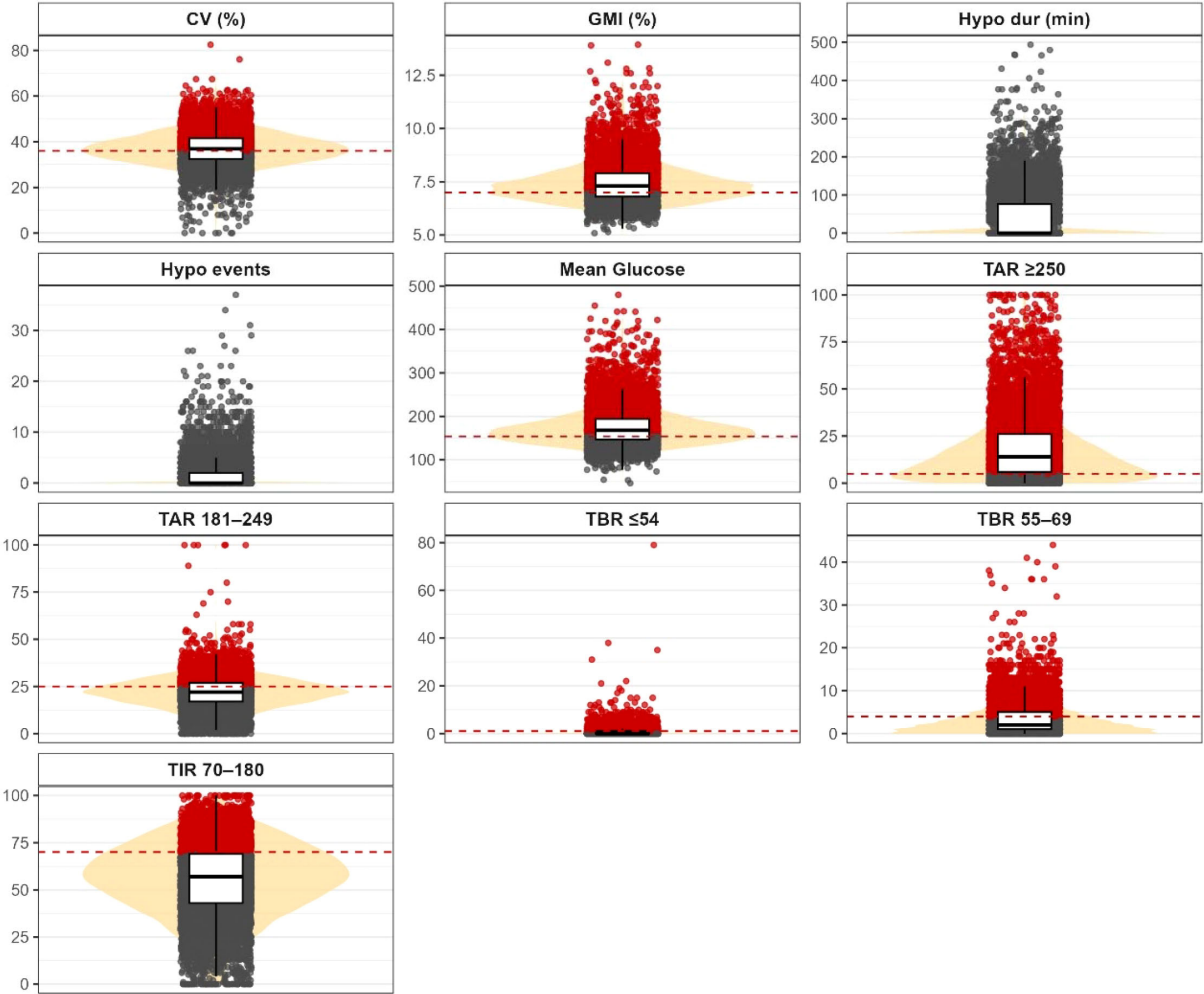
Distribution of CGM metrics with consensus-based clinical targets. Violin and boxplot representations of Intermittently Scanned Continuous Glucose Monitoring (isCGM) metrics. Red dashed horizontal lines indicate the clinical cut-off values recommended by the international ATTD/Battelino consensus for isCGM interpretation. Individual observations exceeding the recommended thresholds are highlighted in red, while values within target remain in grey. MG, Mean Glucose; CV, Coefficient of Variation; GMI, Glucose Management Indicator; TIR, Time in Range; TAR, Time Above Range; TBR, Time Below Range; Hypo dur, Mean Duration of Hypoglycemia Events; Hypo events, Number of Hypoglycemia Events.

A total of 7,885 individuals with T1D were included, of whom 45.4% were women and 54.6% were men (**Table 2**). Mean age was 43.2 years (SD 13.5), with a mean diabetes duration of 20.1 years (SD 12.6). Overall, 12.3% of participants fulfilled the complete AGP target profile, and 24.8% achieved a TIR >70%.From a quantitative perspective, mean time in range (70–180 mg/dL) was 55.4% (SD 19.4), with a mean time spent in hyperglycaemia of 40.3% (SD 20.9) and time in hypoglycaemia <69 mg/dL of 4.2% (SD 5.2). Mean glucose was 175.1 mg/dL (SD 43.2), and mean GMI was 7.45% (SD 0.93) (**Figure 1**). Regarding estimated income level, 57.6% of participants had an annual income below €18,000. The remaining clinical and sociodemographic characteristics of the cohort are shown in **Table 2**.

**Table 2:** Descriptive characteristics of the study population.

Sociodemographic and clinical characteristics		
Sex.		
Male (n, %)	4,252 (5.6)	
Female (n, %)	3,533 (45.4)	
Age, years (mean, SD)	43.2 (13.5)	
18–30 years (n, %)	1,611 (20.69)	
31–45 years (n, %)	2,904 (37.303)	
46–60 years (n, %)	2,422 (31.11)	
61–75 years (n, %)	767 (9.85)	
> 75 years (n,%)	81 (1.04)	
Age at diagnosis, years (mean, SD)	23.2 (14.0)	
0–17 years (n, %)	3,088 (39.7)	
18–30 years (n, %)	2,548 (32.7)	
31–45 years (n, %)	1,562 (20.1)	
46–60 years (n, %)	505 (6.5)	
61–75 years (n, %)	77 (1.0)	
>75 years (n, %)	5 (0.1)	
Diabetes duration, years (mean, SD)	20.1 (12.6)	
<5 years (n, %)	829 (10.6)	
5–9 years (n, %)	1,235 (15,9)	
10–14 years (n, %)	1126 (14,5)	
≥15 years(n, %)	4,595 (59)	
HbA_1c_ % (mean, DE)	7.53 (1.09)	
<6.5%	717 (12.9)	
6.5–7.49%	2,188 (39,5)	
7.5–8.49%	1,735 (31,3)	
8.5%	906 (16,3)	
Estimated income level (%).		
Exempt from co-payment	1,354 (21.6)	
Copayment bracket: <18.000 €	3,609 (57.6)	
Copayment bracket: 18.000–100.000 €	1,170 (18.7)	
Copayment bracket: ≥100.000 €	131 (2.1)	
Glucometric data		
% TIR (mean, DE) (70-180 mg/dl)	55.44 (19.44)	
% TAR I (mean, DE) (181-249 mg/dl)	21.68 (8,71)	
% TAR II (mean, DE) (> 250 mg/dl)	18.64 (17.78)	
% TAR I and II (mean, DE) (>181 mg/dl)	40.32 (20.85)	
% TBR I (mean, DE) (69-55 mg/dl)	3.56 (3.84)	
% TBR II (mean, DE) (< 55 mg/dl)	0.67 (2.42)	
% TBR I and II (mean, DE) (<69 mg/dl)	4.23 (5.22)	
Categorized AGP		
TIR ≥70% (n, %)	Achieved	1,929 (24.8)
	Not achieved	5,856 (75.2)
TAR <25% (n, %)	Achieved	2,721 (35.0)
	Not achieved	5,064 (65.0)
TBR <5% (n, %)	Achieved	5,679 (72.9)
	Not achieved	2,106 (27.1)
Complete AGP profile (n, %)	Achieved	956 (12.3)
	Not achieved	6,829 (87.7)
Other glucometric data		
Coefficient of variation (%) (mean, SD)	37.0 (7.5)	
Mean glucose (mg/dL) (mean, SD)	175.14 (43.18)	
Glucose Management Indicator (GMI) (mean, SD)	7.45 (0.93)	
Mean number of daily sensor scans (mean, SD)	18.09 (24.20)	
Mean duration of hypoglycemic events (min) (mean, SD)	44.29 (62.97)	
Number of hypoglycemic events (mean, SD)	1.49 (2.84)	
Sensor use (%) (mean, SD)	86.5 (21.9)	
Sensor active time (%) (mean, SD)	86.45 (21.94)	
Cumulative days of isCGM use (mean, SD)	746.27 (190.22)	

General description of the cohort included in the study. Variables are presented as mean and standard deviation (SD) or as absolute frequency and percentage (n, %), as appropriate. AGP, Ambulatory Glucose Profile; CV, Coefficient of Variation; GMI, Glucose Management Indicator; HbA1c, Glycated Hemoglobin; MG, Mean Glucose; isCGM, intermittently scanned continuous glucose monitoring; TAR, Time Above Range; TAR I, Time Above Range Level 1; TAR II, Time Above Range Level 2; TBR, Time Below Range; TBR I, Time Below Range Level 1; TBR II, Time Below Range Level 2; TIR, Time in Range.

### 3.2 Sociodemographic variables

Patients who fulfilled the complete AGP target profile were significantly older than those who did not (47.96 vs 42.56 years; *p* < 0.001). Similarly, individuals achieving a TIR >70% were older than those who did not meet this target (45.88 vs 42.35 years; *p* < 0.001) (see **Supplementary Material**).

**Figure 2:**
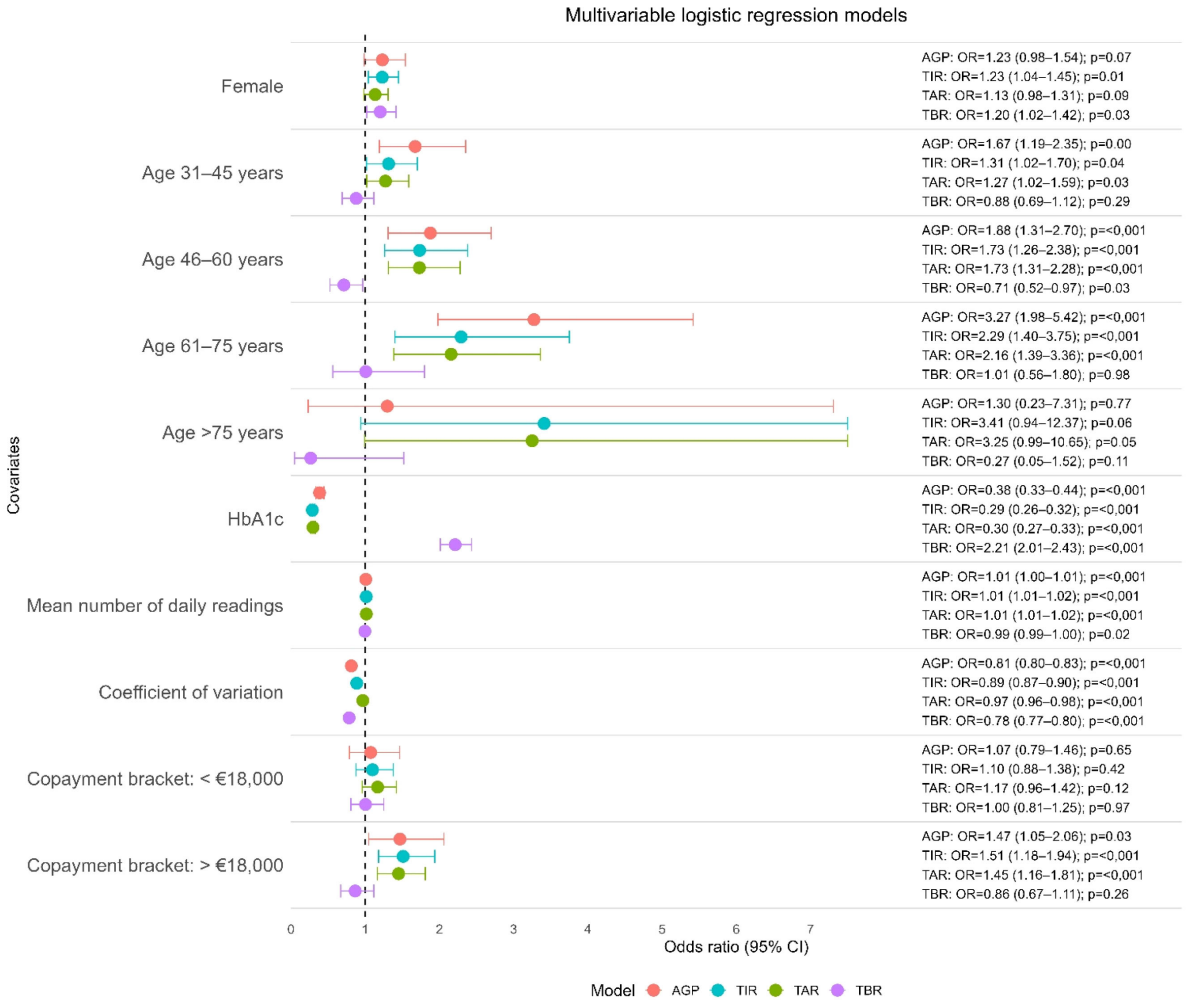
Multivariable logistic regression models for isCGM-derived glycemic. Outcomes Forest plot displaying adjusted odds ratios (ORs) and 95% confidence intervals from multivariable logistic regression models evaluating factors associated with key isCGM-derived glycemic outcomes. Separate models were fitted for AGP metrics, Time in Range (TIR), Time Above Range (TAR), and Time Below Range (TBR), shown in different colors. Each point estimate represents the effect of the corresponding covariate on the likelihood of meeting the recommended CGM target for that metric. Vertical dashed line denotes OR , 1. AGP, Ambulatory Glucose Profile; TIR, Time in Range; TAR, Time Above Range; TBR, Time Below Range; HbA1c, glycated hemoglobin.

In multivariable analyses (**Figure 2**), age remained an independent predictor of both complete AGP target achievement and TIR > 70% after adjustment for the remaining covariates included in the models.

Sex showed differential associations depending on the glycaemic target analysed. In multivariable analysis, male sex was independently associated with achieving TIR > 70% (OR 1.23; 95% CI: 1.04–1.45; *p* = 0.01), whereas female sex was associated with a higher likelihood of achieving the TBR < 4% target (OR 1.20; 95% CI: 1.02–1.42; *p* = 0.03).

Age at diabetes onset was mainly associated with the TBR < 4% target. In the multivariable model, later onset was independently associated with a higher probability of achieving this objective, while no significant associations were observed with TIR, TAR, or complete AGP target achievement.

### 3.3 Glucometric metrics

Patients who fulfilled the complete AGP target profile showed significantly lower HbA_1c_ values (6.90% vs 7.62%; *p* < 0.001), lower mean glucose (138.72 vs 180.24 mg/dL; *p* < 0.001), and lower GMI. They also exhibited lower glycaemic variability, as reflected by a lower coefficient of variation (29.46% vs 38.06%; *p* < 0.001) (see **Supplementary Material**).

Similar findings were observed for the TIR > 70% target, with lower HbA_1c_ (6.87% vs 7.75%; *p* < 0.001), mean glucose (135.57 vs 188.17 mg/dL; *p* < 0.001), and GMI (6.56% vs 7.75%; *p* < 0.001), as well as lower CV (35.49% vs 37.82%; *p* < 0.001).

In multivariable models (**Figure 2**), HbA_1c_ remained an independent predictor of all glycaemic targets analysed. For TIR > 70%, lower HbA_1c_ was associated with a higher likelihood of target achievement (OR 0.29; 95% CI: 0.26–0.32; *p* < 0.001). Comparable results were observed for complete AGP target achievement and TAR < 25%.

Glycaemic variability was also independently associated with all outcomes, with lower CV values being associated with a higher probability of achieving complete AGP target, TIR > 70%, TAR < 25%, and TBR < 4%.

The number of daily sensor scans was higher among patients achieving the complete AGP target (27.66 vs 16.75; *p* < 0.001) and TIR > 70% (26.08 vs 15.46; *p* < 0.001), and remained an independent predictor in the corresponding multivariable models.

### 3.4 Socioeconomic status

Estimated income level, assessed using the pharmaceutical co-payment bracket, was significantly associated with several glycaemic control targets.

In univariate analysis (see **Supplementary Material**), the intermediate income bracket (€18,000–€100,000/year) showed a higher likelihood of achieving the complete AGP target profile (OR 1.52; 95% CI: 1.22–1.91; *p* < 0.001). This association remained significant in multivariable analysis (OR 1.47; 95% CI: 1.05–2.06; *p* = 0.03).

For the TIR > 70% target, achievement was more frequent in the intermediate (€18,000–€100,000/year) and high (> €100,000/year) income categories, with a particularly strong association for the latter in univariate analysis (OR 2.20; 95% CI: 1.51–3.21; *p* < 0.001). In the multivariable model (**Figure 2**), only the intermediate income bracket remained an independent predictor (OR 1.51; 95% CI: 1.18–1.94; *p* < 0.001).

Similarly, achievement of the TAR < 25% target was associated with both the intermediate (€18,000–€100,000/year) (OR 1.92; 95% CI: 1.63–2.26; *p* < 0.001) and high (> €100,000/year) (OR 2.37; 95% CI: 1.65–3.41; *p* < 0.001) income categories in univariate analysis. After multivariable adjustment, the association remained significant only for the intermediate income bracket (OR 1.45; 95% CI: 1.16–1.81; *p* < 0.001).

In contrast, no significant associations were observed between income level and achievement of the TBR < 4% target in multivariable analysis.

A complete graphical representation of the multivariable analysis using forest plots is shown in **Figure 2**.

## 4 Discussion

This population-based study identifies clinical and sociodemographic factors associated with the achievement of glycaemic control targets in adults with T1D using isCGM.

Our findings show a consistent association between HbA_1c_ values and AGP-derived metrics, whereby lower HbA_1c_ levels were correlated with higher TIR, reduced exposure to hyperglycaemia, and lower glycaemic variability, as expected. This concordance between a traditional marker of metabolic control and monitoring-derived glucometric parameters reinforces the internal validity of the models and supports the robustness of the interpretation of the remaining associations identified (15). Although HbA_1c_ remains a fundamental marker, its inability to reflect daily glycaemic variability and the risk of acute dysglycaemic events highlights the need to interpret it alongside dynamic metrics such as TIR and CV (2, 4, 16, 17), thereby justifying their role as independent predictors in our models (3, 18).

The association between income level and achievement of TIR, TAR, and complete AGP targets constitutes one of the most relevant findings of this study. Participants in the middle-to-high income categories (> €18,000/year) showed a higher probability of achieving these objectives, indicating that social inequalities persist even when economic access to glucose monitoring technology is guaranteed (9). This pattern is consistent with previous literature documenting the relationship between socioeconomic status, glycaemic control, and diabetes-related complications in T1D (19, 20). Our results suggest that, even in a setting of universal isCGM coverage, an income-related gap remains that may influence the extent to which the technology is effectively leveraged in clinical practice. This underscores the need to implement targeted support and optimization strategies for lower-income groups, with the aim of reducing inequalities in glycaemic control and maximizing the real-world impact of this technology (21).

Regarding demographic variables, the association between older age and better glycaemic control may reflect more stable adherence patterns and a more conservative approach to treatment management (22).

The association between male sex and achievement of TIR > 70% remains insufficiently explained, and no studies have specifically demonstrated a consistent relationship between TIR and sex. However, sex-related differences in CGM-related outcomes and diabetes-related complications have been reported (23). Population-based studies have shown differences in acute complications and their response to CGM implementation according to sex (12, 13), while registry data suggest gender-related differences in self-management and complications despite similar overall glycaemic control (24). In addition, sex-related differences in psychological burden and perception of glycaemic variability have been described, with women showing higher levels of anxiety, depression and greater impact of glycaemic excursions (25). These factors may influence CGM-derived metrics through behavioural and psychosocial pathways, although the underlying mechanisms remain poorly understood.

In contrast, achievement of the hypoglycaemia target (TBR < 4%) was mainly related to individual clinical variables—such as later age at T1D diagnosis or higher HbA_1c_ —and not to socioeconomic status. This finding differs from that reported in recent studies focusing on severe hypoglycaemia and impaired awareness of hypoglycaemia, where socioeconomic deprivation and psychological factors have been identified as relevant risk determinants (26). This discrepancy likely reflects differences in what these measures capture. isCGM-derived metrics such as TBR quantify overall exposure to low glucose levels, whereas severe or unrecognised hypoglycaemia represents a more complex clinical phenomenon that is not fully captured by percentage-based measures of time spent in hypoglycaemia (27).

The low proportion of participants achieving the complete AGP target profile (12.3%) highlights the demanding nature of current consensus-based criteria. This finding is in line with previous studies reporting difficulties in simultaneously achieving all recommended metrics, even in cohorts with broad access to isCGM and structured diabetes education programmes (28–30).

Several limitations of this study should be acknowledged. The cross-sectional design precludes the establishment of causal relationships. Additionally, the exclusion of individuals with isCGM integrated into the electronic health record but insufficient data for analysis may have introduced selection bias. These patients may exhibit lower adherence to technology use, a greater digital divide, or other unmeasured characteristics, potentially limiting the generalisability of the results to the entire population of isCGM users with T1D. The pharmaceutical co-payment bracket represents an indirect proxy of socioeconomic status and does not capture key dimensions such as educational level or social support. Furthermore, the exclusion of continuous subcutaneous insulin infusion (CSII) users limits the extrapolation of these associations factors to this form of intensive insulin therapy; however, the use of CSII outside closed-loop systems is progressively declining due to the advantages of automated insulin delivery (31, 32), reducing the practical relevance of identifying predictors of glycaemic control in CSII users relying solely on isCGM. Finally, emerging metrics such as narrow TIR or the Glycaemic Risk Index—currently proposed as complementary tools for characterising glycaemic risk (33–36)—were not included and warrant evaluation in future studies.

Among the strengths of this study are the large and representative sample size, the use of centralised and standardised data from the Andalusian Public Health System, and the assessment of glycaemic control using AGP-derived metrics based on international consensus criteria (5). The incorporation of a socioeconomic dimension represents a novel contribution, as the interaction between income level and isCGM-based glycaemic control has been insufficiently explored to date.

Overall, this study shows that clinical, sociodemographic, and glucometric factors are independently associated with the level of glycaemic control achieved by adults with T1D in a healthcare system with universal access to interstitial glucose monitoring. The size of the cohort and the consistency of the findings support the complementary value of isCGM-derived metrics and HbA_1c_ for characterising metabolic control profiles in real-world clinical practice.

In conclusion, this study identifies clinical and social factors associated with achieving glycaemic outcomes and highlights glucometric variables and indicators of isCGM use as relevant correlates of glycaemic control in adults with T1D treated with MDI. These findings have important clinical and population-level implications and support the potential value of targeted strategies to optimise the use of diabetes technology in socially vulnerable populations.

## Data Availability

The datasets presented in this study can be found in online repositories. The names of the repository/repositories and accession number(s) can be found in the article/**Supplementary Material**.
