# Supplementary material for: Clinical and socioeconomic determinants of glycaemic control derived from continuous glucose monitoring in adults with type 1 diabetes
Source: Front Endocrinol (Lausanne). 2026 Apr 23;17:1813510. doi: 10.3389/fendo.2026.1813510 (PMC13149104; doi:10.3389/fendo.2026.1813510)
Supplement: Supplementary file 1 [file DataSheet1.docx]

Supplementary Material 1:


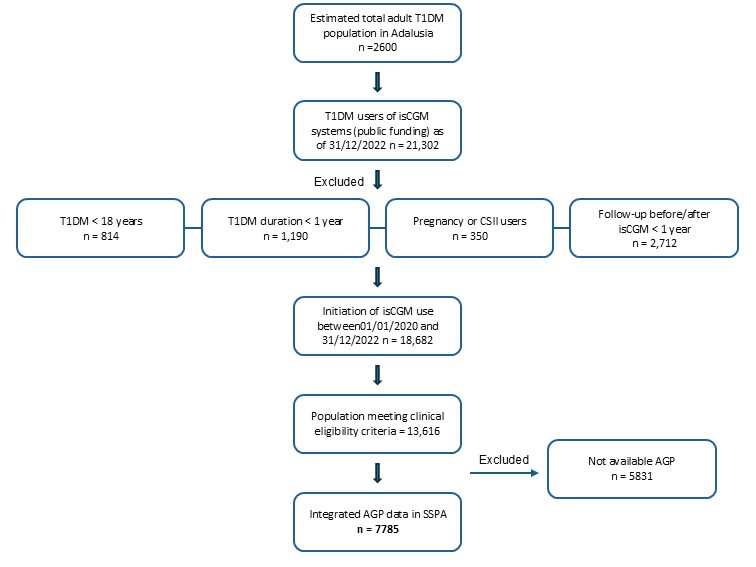


**Supplementary Figure 1. Flow diagram of participant eligibility and study population selection.**

Flowchart illustrating the selection of the study population from the adult type 1 diabetes mellitus (T1DM) population in Andalusia. Adults using intermittently scanned continuous glucose monitoring (isCGM) with public funding were identified. Exclusion criteria included age <18 years, diabetes duration <1 year, pregnancy, use of continuous subcutaneous insulin infusion (CSII), and insufficient isCGM follow-up. The final study population consisted of individuals with integrated ambulatory glucose profile (AGP) data available in the Andalusian Public Health System electronic health records.

**Supplementary Material 2: Description of estimated income level**

The estimated income level of participants was obtained from the Population Health Database, using the pharmaceutical co-payment bracket as a proxy variable. This bracket is automatically assigned according to officially declared annual income, employment or pensioner status, and other criteria defined in the current regulatory framework of the Spanish National Health System. This nationwide standardized classification identifies whether individuals are exempt from pharmaceutical co-payment or contribute 10%, 40%, 50%, or 60% of medication costs, with different monthly caps depending on income level. As such, it provides an objective and routinely recorded indicator of socioeconomic status within the public healthcare system. Based on this classification, participants were grouped into three income categories: < €18,000/year, €18,000–€100,000/year, and > €100,000/year, in accordance with the official documentation of the Spanish Ministry of Health. <https://www.sanidad.gob.es/estadEstudios/estadisticas/sisInfSanSNS/pdf/01.01.2024_aportacion_al_pago_medicamentos_por_receta_SNS.pdf>

**Supplementary Material 3. Statistical analysis.**

*Univariate analysis*

| Variable | AGP (0) | AGP (1) | Difference | p value |
| --- | --- | --- | --- | --- |
| Male | 3,735 / 87.8% | 517 / 12.2% | ref |  |
| Female | 3,094 / 87.6% | 439 / 12.4% | 1.03 [0.89; 1.17] | 0.7211 |
| Age (years) | 42.56 ± 13.25 | 47.96 ± 13.95 | 5.39 [4.46; 6.33] | 0.0000 |
| 18–30 years | 1487 / 92.3% | 124 / 7.7% | Ref. |  |
| 31–45 years | 2,611 / 89.9% | 293 / 10.1% | 1.35 [1.08; 1.68] | 0.0080 |
| 46–60 years | 2,084 / 86.0% | 338 / 14.0% | 1.94 [1.57; 2.41] | 0.0000 |
| 61–75 years | 584 / 76.1% | 183 / 23.9% | 3.76 [2.93; 4.81] | 0.0000 |
| >75 years | 63 / 77.8% | 18 / 22.2% | 3.43 [1.97; 5.97] | 0.0000 |
| Age at onset (years) | 22.68 ± 13.72 | 26.66 ± 15.11 | 3.98 [2.97; 4.99] | 0.0000 |
| 0–17 years | 2,784 / 90.2% | 304 / 9.8% | Ref. |  |
| 18–30 years | 2,252 / 88.4% | 296 / 11.6% | 1.20 [1.02; 1.43] | 0.0320 |
| 31–45 years | 1,336 / 85.5% | 226 / 14.5% | 1.55 [1.29; 1.86] | 0.0000 |
| 46–60 years | 392 / 77.6% | 113 / 22.4% | 2.64 [2.08; 3.36] | 0.0000 |
| 61–75 years | 65 / 79.3% | 17 / 20.7% | 2.40 [1.39; 4.14] | 0.0017 |
| Diabetes duration (years) | 19.88 ± 12.39 | 21.29 ± 14.18 | 1.41 [0.47; 2.36] | 0.0035 |
| <5 years | 705 / 85.0% | 124 / 15.0% | Ref. |  |
| 5–9 years | 1,098 / 88.9% | 137 / 11.1% | 0.71 [0.55; 0.92] | 0.0098 |
| 10–14 years | 990 / 87.9% | 136 / 12.1% | 0.78 [0.60; 1.01] | 0.0643 |
| ≥15 years | 4,036 / 87.8% | 559 / 12.2% | 0.79 [0.64; 0.97] | 0.0260 |
| Time in range (%) (70–180 mg/dL) | 51.74 ± 17.67 | 81.86 ± 7.35 | 30.12 [29.49; 30.75] | 0.0000 |
| Time above range I (%) (181–249 mg/dL) | 22.74 ± 8.51 | 14.15 ± 6.04 | -8.58 [-9.02; -8.15] | 0.0000 |
| Time above range II (%) (≥250 mg/dL) | 20.95 ± 17.80 | 2.16 ± 1.62 | -18.79 [-19.22; -18.35] | 0.0000 |
| Time below range I (%) (55–69 mg/dL) | 3.82 ± 4.00 | 1.76 ± 1.36 | -2.06 [-2.18; -1.93] | 0.0000 |
| Time below range II (%) (≤54 mg/dL) | 0.76 ± 2.57 | 0.07 ± 0.25 | -0.69 [-0.75; -0.63] | 0.0000 |
| Total time above range (%) (>180 mg/dL) | 43.68 ± 19.90 | 16.31 ± 7.16 | -27.37 [-28.03; -26.72] | 0.0000 |
| Total time below range (%) (<70 mg/dL) | 4.57 ± 5.46 | 1.83 ± 1.43 | -2.75 [-2.90; -2.59] | 0.0000 |
| HbA_1c_ (%) | 7.62 ± 1.10 | 6.90 ± 0.73 | -0.72 [-0.78; -0.65] | 0.0000 |
| Mean glucose (mg/dL) | 180.24 ± 43.55 | 138.72 ± 11.12 | -41.52 [-42.77; -40.27] | 0.0000 |
| Number of hypoglycemic events | 1.64 ± 2.96 | 0.42 ± 1.34 | -1.21 [-1.32; -1.10] | 0.0000 |
| Mean duration of hypoglycemic events (min) | 48.32 ± 64.84 | 15.52 ± 36.29 | -32.80 [-35.57; -30.03] | 0.0000 |
| Glucose Management Indicator (GMI, %) | 7.56 ± 0.93 | 6.64 ± 0.25 | -0.92 [-0.95; -0.89] | 0.0000 |
| Coefficient of variation (%) | 38.06 ± 7.12 | 29.46 ± 5.06 | -8.60 [-8.96; -8.24] | 0.0000 |
| Mean number of daily sensor scans | 16.75 ± 19.23 | 27.66 ± 45.02 | 10.91 [8.02; 13.80] | 0.0000 |
| Sensor active time (%) | 86.12 ± 21.89 | 88.79 ± 22.15 | 2.66 [1.17; 4.16] | 0.0005 |
| Cumulative days of isCGM use | 747.01 ± 189.58 | 741.01 ± 194.73 | -6.00 [-19.14; 7.14] | 0.3710 |
| Co-payment bracket: exempt | 1,197 / 88.4% | 157 / 11.6% | Ref. |  |
| Co-payment bracket: <€18,000/year | 3,277 / 90.8% | 332 / 9.2% | 0.77 [0.63; 0.94] | 0.0118 |
| Co-payment bracket: €18,000–€100,000/year | 975 / 83.3% | 195 / 16.7% | 1.52 [1.22; 1.91] | 0.0003 |
| Co-payment bracket: ≥ €100,000/year | 113 / 86.3% | 18 / 13.7% | 1.21 [0.72; 2.05] | 0.4678 |

***Table 3.1: Univariate analysis of achievement of the AGP glycemic target.***

*Comparison of clinical, sociodemographic, and glucometric variables between participants who did and did not achieve the AGP glycemic target. AGP (0) indicates failure to achieve the target, whereas AGP (1) indicates target achievement. Differences in quantitative variables are expressed as mean differences, and associations for categorical variables are expressed as odds ratios. In both cases, 95% confidence intervals are reported.*

| Variable | TIR (0) | TIR (1) | Difference | p value |
| --- | --- | --- | --- | --- |
| Male | 3,202 / 75.3% | 1,050 / 24.7% |  |  |
| Female | 2,654 / 75.1% | 879 / 24.9% | 1.01 [0.91; 1.20] | 0.8500 |
| Age (years) | 42.35 ± 13.30 | 45.88 ± 13.56 | 3.54 [2.84; 4.23] | 0.0000 |
| 18–30 years | 1,309 / 81.3% | 302 / 18.7% | Ref. |  |
| 31–45 years | 2,247 / 77.4% | 657 / 22.6% | 1.27 [1.09; 1.48] | 0.0023 |
| 46–60 years | 1,752 / 72.3% | 670 / 27.7% | 1.66 [1.42; 1.93] | 0.0000 |
| 61–75 years | 495 / 64.5% | 272 / 35.5% | 2.38 [1.96; 2.89] | 0.0000 |
| >75 years | 53 / 65.4% | 28 / 34.6% | 2.29 [1.42; 3.68] | 0.0006 |
| Age at onset (years) | 2,389 / 77.4% | 699 / 22.6% | Ref. |  |
| 0–17 years | 1,927 / 75.6% | 621 / 24.4% | 1.10 [0.97; 1.25] | 0.1257 |
| 18–30 years | 1,147 / 73.4% | 415 / 26.6% | 1.24 [1.07; 1.42] | 0.0030 |
| 31–45 years | 333 / 65.9% | 172 / 34.1% | 1.77 [1.44; 2.16] | 0.0000 |
| 46–60 years | 60 / 73.2% | 22 / 26.8% | 1.25 [0.76; 2.06] | 0.3722 |
| 61–75 years | 2,389 / 77.4% | 699 / 22.6% | Ref. |  |
| Diabetes duration (years) | 19.67 ± 12.22 | 21.22 ± 13.73 | 1.56 [0.87; 2.24] | 0.0000 |
| <5 years | 591 / 71.3% | 238 / 28.7% | Ref. |  |
| 5–9 years | 960 / 77.7% | 275 / 22.3% | 0.71 [0.58; 0.87] | 0.0009 |
| 10–14 years | 883 / 78.4% | 243 / 21.6% | 0.68 [0.56; 0.84] | 0.0003 |
| ≥15 years | 3,422 / 74.5% | 1,173 / 25.5% | 0.85 [0.72; 1.00] | 0.0548 |
| Time in range (%) (70–180 mg/dL) | 47.66 ± 15.56 | 79.05 ± 7.07 | 31.39 [30.88; 31.90] | 0.0000 |
| Time above range I (%) (181–249 mg/dL) | 24.41 ± 7.71 | 13.40 ± 5.88 | -11.01 [-11.34; -10.68] | 0.0000 |
| Time above range II (%) (≥250 mg/dL) | 23.82 ± 17.61 | 2.92 ± 2.47 | -20.90 [-21.36; -20.43] | 0.0000 |
| Time below range I (%) (55–69 mg/dL) | 3.37 ± 3.82 | 4.14 ± 3.82 | 0.77 [0.58; 0.97] | 0.0000 |
| Time below range II (%) (≤54 mg/dL) | 0.73 ± 2.72 | 0.48 ± 1.11 | -0.25 [-0.34; -0.17] | 0.0000 |
| Total time above range (%) (>180 mg/dL) | 48.23 ± 17.52 | 16.32 ± 7.46 | -31.91 [-32.47; -31.35] | 0.0000 |
| Total time below range (%) (<70 mg/dL) | 4.11 ± 5.42 | 4.63 ± 4.53 | 0.52 [0.28; 0.77] | 0.0000 |
| HbA_1c_ (%) | 7.75 ± 1.08 | 6.87 ± 0.81 | -0.88 [-0.94; -0.83] | 0.0000 |
| Mean glucose (mg/dL) | 188.17 ± 41.59 | 135.57 ± 13.89 | -52.60 [-53.83; -51.37] | 0.0000 |
| Number of hypoglycemic events | 1.49 ± 2.77 | 1.47 ± 3.05 | -0.02 [-0.17; 0.14] | 0.8185 |
| Mean duration of hypoglycemic events (min) | 45.59 ± 64.18 | 40.36 ± 58.98 | -5.23 [-8.34; -2.13] | 0.0010 |
| Glucose Management Indicator (GMI, %) | 7.75 ± 0.88 | 6.56 ± 0.32 | -1.18 [-1.21; -1.15] | 0.0000 |
| Coefficient of variation (%) | 1.49 ± 2.77 | 1.47 ± 3.05 | -0.02 [-0.17; 0.14] | 0.8185 |
| Mean number of daily sensor scans | 15.46 ± 15.81 | 26.08 ± 39.00 | 10.62 [8.84; 12.41] | 0.0000 |
| Sensor active time (%) | 85.44 ± 22.26 | 89.52 ± 20.65 | 4.08 [3.00; 5.17] | 0.0000 |
| Cumulative days of isCGM use | 743.54 ± 190.40 | 754.55 ± 189.46 | 11.01 [1.25; 20.77] | 0.0271 |
| Co-payment bracket: exempt | 1,065 / 78.7% | 289 / 21.3% | Ref. |  |
| Co-payment bracket: <€18,000/year | 2,851 / 79.0% | 758 / 21.0% | 0.98 [0.84; 1.14] | 0.7931 |
| Co-payment bracket: €18,000–€100,000/year | 780 / 66.7% | 390 / 33.3% | 1.84 [1.54; 2.20] | 0.0000 |
| Co-payment bracket: ≥ €100,000/year | 82 / 62.6% | 49 / 37.4% | 2.20 [1.51; 3.21] | 0.0000 |

***Table 3.2. Univariate analysis of achievement of the TIR glycemic target***

*Comparison of clinical, sociodemographic, and glucometric variables between participants who did and did not achieve the TIR glycemic target. TIR (0) indicates failure to achieve the target, whereas TIR (1) indicates target achievement. Differences in quantitative variables are expressed as mean differences, and associations for categorical variables are expressed as odds ratios. In both cases, 95% confidence intervals are reported.*

| Variable | TAR (0) | TAR (1) | Difference | p value |
| --- | --- | --- | --- | --- |
| Male | 2,751 / 64.7% | 1,501 / 35.3% |  |  |
| Female | 2,313 / 65.5% | 1,220 / 34.5% | 0.97 [0.88; 1.06] | 0.4784 |
| Age (years) | 42.46 ± 13.48 | 44.65 ± 13.29 | 2.19 [1.57; 2.81] | 0.0000 |
| 18–30 years | 1,126 / 69.9% | 485 / 30.1% | Ref. |  |
| 31–45 years | 1,943 / 66.9% | 961 / 33.1% | 1.15 [1.01; 1.31] | 0.0394 |
| 46–60 years | 1,497 / 61.8% | 925 / 38.2% | 1.43 [1.25; 1.64] | 0.0000 |
| 61–75 years | 446 / 58.1% | 321 / 41.9% | 1.67 [1.40; 2.00] | 0.0000 |
| >75 years | 52 / 64.2% | 29 / 35.8% | 1.29 [0.81; 2.06] | 0.2778 |
| Age at onset (years) | 22.95 ± 13.96 | 23.58 ± 13.95 | 0.63 [-0.02; 1.28] | 0.0589 |
| 0–17 years | 2,040 / 66.1% | 1,048 / 33.9% | Ref. |  |
| 18–30 years | 1,647 / 64.6% | 901 / 35.4% | 1.06 [0.95; 1.19] | 0.2636 |
| 31–45 years | 1,016 / 65.0% | 546 / 35.0% | 1.05 [0.92; 1.19] | 0.4900 |
| 46–60 years | 307 / 60.8% | 198 / 39.2% | 1.26 [1.03; 1.52] | 0.0212 |
| 61–75 years | 54 / 65.9% | 28 / 34.1% | 1.01 [0.64; 1.60] | 0.9686 |
| Diabetes duration (years) | 19.51 ± 12.21 | 21.07 ± 13.32 | 1.56 [0.96; 2.16] | 0.0000 |
| <5 years | 515 / 62.1% | 314 / 37.9% | Ref. |  |
| 5–9 years | 854 / 69.1% | 381 / 30.9% | 0.73 [0.61; 0.88] | 0.0009 |
| 10–14 years | 769 / 68.3% | 357 / 31.7% | 0.76 [0.63; 0.92] | 0.0045 |
| ≥15 years | 2,926 / 63.7% | 1,669 / 36.3% | 0.94 [0.80; 1.09] | 0.3923 |
| Time in range (%) (70–180 mg/dL) | 45.05 ± 14.98 | 74.77 ± 9.49 | 29.72 [29.17; 30.26] | 0.0000 |
| Time above range I (%) (181–249 mg/dL) | 25.46 ± 7.55 | 14.65 ± 5.91 | -10.81 [-11.11; -10.50] | 0.0000 |
| Time above range II (%) (≥250 mg/dL) | 26.40 ± 17.54 | 4.19 ± 3.38 | -22.21 [-22.70; -21.71] | 0.0000 |
| Time below range I (%) (55–69 mg/dL) | 2.62 ± 2.82 | 5.32 ± 4.75 | 2.70 [2.50; 2.89] | 0.0000 |
| Time below range II (%) (≤54 mg/dL) | 0.46 ± 1.12 | 1.06 ± 3.77 | 0.60 [0.45; 0.74] | 0.0000 |
| Total time above range (%) (>180 mg/dL) | 51.86 ± 15.88 | 18.85 ± 8.06 | -33.01 [-33.54; -32.48] | 0.0000 |
| Total time below range (%) (<70 mg/dL) | 3.08 ± 3.56 | 6.38 ± 6.87 | 3.30 [3.02; 3.57] | 0.0000 |
| HbA_1c_ (%) | 7.86 ± 1.08 | 6.93 ± 0.80 | -0.93 [-0.98; -0.88] | 0.0000 |
| Mean glucose (mg/dL) | 195.34 ± 39.83 | 137.53 ± 14.46 | -57.81 [-59.03; -56.58] | 0.0000 |
| Number of hypoglycemic events | 1.10 ± 2.12 | 2.20 ± 3.73 | 1.10 [0.95; 1.25] | 0.0000 |
| Mean duration of hypoglycemic events (min) | 38.51 ± 60.24 | 55.05 ± 66.44 | 16.54 [13.54; 19.54] | 0.0000 |
| Glucose Management Indicator (GMI, %) | 7.90 ± 0.83 | 6.61 ± 0.32 | -1.29 [-1.32; -1.26] | 0.0000 |
| Coefficient of variation (%) | 37.82 ± 7.47 | 35.49 ± 7.18 | -2.33 [-2.67; -1.99] | 0.0000 |
| Mean number of daily sensor scans | 15.04 ± 15.73 | 23.77 ± 34.16 | 8.73 [7.38; 10.09] | 0.0000 |
| Sensor active time (%) | 85.09 ± 22.33 | 88.97 ± 20.97 | 3.88 [2.88; 4.88] | 0.0000 |
| Cumulative days of isCGM use | 741.39 ± 192.09 | 755.36 ± 186.37 | 13.97 [5.20; 22.75] | 0.0018 |
| Co-payment bracket: exempt | 957 / 70.7% | 397 / 29.3% | Ref. |  |
| Co-payment bracket: <€18,000/year | 2458 / 68.1% | 1151 / 31.9% | 1.13 [0.98; 1.29] | 0.082 |
| Co-payment bracket: €18,000–€100,000/year | 651 / 55.6% | 519 / 44.4% | 1.92 [1.63; 2.26] | 0.0000 |
| Co-payment bracket: ≥ €100,000/year | 66 / 50.4% | 65 / 49.6% | 2.37 [1.65; 3.41] | 0.0000 |

***Table 3.3. Univariate analysis of achievement of the TAR glycemic target***

*Comparison of clinical, sociodemographic, and glucometric variables between participants who did and did not achieve the TAR glycemic target. TAR (0) indicates failure to achieve the target, whereas TAR (1) indicates target achievement. Differences in quantitative variables are expressed as mean differences, and associations for categorical variables are expressed as odds ratios. In both cases, 95% confidence intervals are reported.*

| Variable | TBR (0) | TBR (1) | Difference | p value |
| --- | --- | --- | --- | --- |
| Male | 1233 / 29.00% | 3019 / 71,00% |  |  |
| Female | 873 / 24,70% | 2660 / 75,29% | 1,24 [1,13;1,37] | 0,0000 |
| Age (years) | 40.93 ± 12.06 | 44.07 ± 13.84 | 3.14 [2.51; 3.77] | 0.0000 |
| 18–30 years | 496 / 30.8% | 1115 / 69.2% | Ref. |  |
| 31–45 years | 868 / 29.9% | 2036 / 70.1% | 1.04 [0.91; 1.19] | 0.529 |
| 46–60 years | 628 / 25.9% | 1794 / 74.1% | 1.27 [1.11; 1.46] | 0.0000 |
| 61–75 years | 105 / 13.7% | 662 / 86.3% | 2.80 [2.23; 3.54] | 0.0000 |
| >75 years | 9 / 11.1% | 72 / 88.9% | 3.56 [1.77; 7.17] | 0.0000 |
| Age at onset (years) | 20.70 ± 12.62 | 24.09 ± 14.32 | 3.39 [2.73; 4.04] | 0.0000 |
| 0–17 years | 959 / 31.1% | 2129 / 68.9% | Ref. |  |
| 18–30 years | 713 / 28.0% | 1835 / 72.0% | 1.16 [1.03; 1.30] | 0.0120 |
| 31–45 years | 343 / 22.0% | 1219 / 78.0% | 1.60 [1.39; 1.85] | 0.0000 |
| 46–60 years | 82 / 16.2% | 423 / 83.8% | 2.32 [1.81; 2.98] | 0.0000 |
| 61–75 years | 9 / 11.0% | 73 / 89.0% | 3.65 [1.82; 7.33] | 0.0003 |
| Diabetes duration (years) | 20.23 ± 12.17 | 19.99 ± 12.80 | -0.25 [-0.86; 0.37] | 0.4345 |
| <5 years | 205 / 24.7% | 624 / 75.3% | Ref. |  |
| 5–9 years | 318 / 25.7% | 917 / 74.3% | 0.95 [0.77; 1.16] | 0.6013 |
| 10–14 years | 281 / 25.0% | 845 / 75.0% | 0.99 [0.80; 1.22] | 0.9086 |
| ≥15 years | 1,302 / 28.3% | 3293 / 71.7% | 0.83 [0.70; 0.99] | 0.0330 |
| Time in range (%) (70–180 mg/dL) | 60.61 ± 14.58 | 53.53 ± 20.64 | -7.08 [-7.90; -6.26] | 0.0000 |
| Time above range I (%) (181–249 mg/dL) | 16.92 ± 6.72 | 23.45 ± 8.70 | 6.53 [6.17; 6.90] | 0.0000 |
| Time above range II (%) (≥250 mg/dL) | 11.95 ± 10.99 | 21.12 ± 19.13 | 9.16 [8.48; 9.85] | 0.0000 |
| Time below range I (%) (55–69 mg/dL) | 8.38 ± 4.06 | 1.78 ± 1.51 | -6.60 [-6.78; -6.43] | 0.0000 |
| Time below range II (%) (≤54 mg/dL) | 2.14 ± 4.29 | 0.13 ± 0.37 | -2.01 [-2.20; -1.83] | 0.0000 |
| Total time above range (%) (>180 mg/dL) | 28.87 ± 15.12 | 44.57 ± 21.08 | 15.70 [14.85; 16.54] | 0.0000 |
| Total time below range (%) (<70 mg/dL) | 10.52 ± 6.28 | 1.90 ± 1.62 | -8.62 [-8.89; -8.35] | 0.0000 |
| HbA_1c_ (%) | 7.22 ± 1.03 | 7.64 ± 1.09 | 0.42 [0.36; 0.48] | 0.0000 |
| Mean glucose (mg/dL) | 150.37 ± 28.73 | 184.32 ± 44.03 | 33.95 [32.27; 35.63] | 0.0000 |
| Number of hypoglycemic events | 4.06 ± 4.03 | 0.54 ± 1.30 | -3.52 [-3.70; -3.34] | 0.0000 |
| Mean duration of hypoglycemic events (min) | 101.88 ± 67.46 | 22.98 ± 45.46 | -78.91 [-82.03; -75.79] | 0.0000 |
| Glucose Management Indicator (GMI, %) | 6.90 ± 0.65 | 7.65 ± 0.94 | 0.75 [0.71; 0.79] | 0.0000 |
| Coefficient of variation (%) | 42.82 ± 6.73 | 34.85 ± 6.49 | -7.98 [-8.31; -7.64] | 0.0000 |
| Mean number of daily sensor scans | 17.07 ± 22.34 | 18.47 ± 24.85 | 1.39 [0.24; 2.55] | 0.0180 |
| Sensor active time (%) | 85.91 ± 21.60 | 86.65 ± 22.06 | 0.74 [-0.35; 1.82] | 0.1839 |
| Cumulative days of isCGM use | 757.25 ± 184.08 | 742.20 ± 192.30 | -15.05 [-24.37; -5.74] | 0.0016 |
| Co-payment bracket: exempt | 323 / 23.9% | 1031 / 76.1% | Ref. |  |
| Co-payment bracket: <€18,000/year | 1,062 / 29.4% | 2,547 / 70.6% | 0.75 [0.65; 0.87] | 0.0001 |
| Co-payment bracket: €18,000–€100,000/year | 331 / 28.3% | 839 / 71.7% | 0.79 [0.66; 0.95] | 0.0113 |
| Co-payment bracket: ≥ €100,000/year | 39 / 29.8% | 92 / 70.2% | 0.74 [0.50; 1.10] | 0.1333 |

***Table 3.4. Univariate analysis of achievement of the TBR glycemic target***

*Comparison of clinical, sociodemographic, and glucometric variables between participants who did and did not achieve the TBR glycemic target. TBR (0) indicates failure to achieve the target, whereas TBR (1) indicates target achievement. Differences in quantitative variables are expressed as mean differences, and associations for categorical variables are expressed as odds ratios. In both cases, 95% confidence intervals are reported.*

*Multivariable analysis:*

| Variable | OR | 95% CI lower limit | 95% CI upper limit | p value |
| --- | --- | --- | --- | --- |
| Male | 1.231 | 0.985 | 1.540 | 0.068 |
| Age 31–45 years | 1.673 | 1.189 | 2.353 | 0.003 |
| Age 46–60 years | 1.878 | 1.308 | 2.697 | <0.001 |
| Age 61–75 years | 3.274 | 1.978 | 5.418 | <0.001 |
| >75 years | 1.297 | 0.230 | 7.308 | 0.768 |
| HbA_1c_ (%) | 0.385 | 0.334 | 0.443 | <0.001 |
| Mean number of daily sensor scans | 1.007 | 1.003 | 1.011 | <0.001 |
| Coefficient of variation (%) | 0.813 | 0.796 | 0.829 | <0.001 |
| Co-payment bracket: < €18,000/year | 1.074 | 0.788 | 1.463 | 0.653 |
| Co-payment bracket: ≥ €18,000/year | 1.468 | 1.047 | 2.060 | 0.026 |

Goodness of fit (H. Lemeshow) 32.63, p<0.001; AUC: 0.89 [0.877; 0.902], p<0.001

***Table 3.5. Multivariable logistic regression model for achievement of glycemic control targets: complete AGP.***

*Independent factors associated with achievement of the complete AGP glycemic target. Adjusted odds ratios (ORs) with 95% confidence intervals (95% CIs) and p values are reported. Model discrimination was assessed using the area under the receiver operating characteristic curve (AUC), and calibration was evaluated using the Hosmer–Lemeshow goodness-of-fit test. The co-payment categories “€18,000–€100,000/year” and “≥ €100,000/year” were combined due to the small sample size of the highest income bracket, in order to improve the robustness of the estimates.*

| Variable | OR | 95% CI lower limit | 95% CI upper limit | p value |
| --- | --- | --- | --- | --- |
| Male | 1.229 | 1.043 | 1.449 | 0.014 |
| Age 31–45 years | 1.315 | 1.017 | 1.701 | 0.037 |
| Age 46–60 years | 1.733 | 1.262 | 2.379 | <0.001 |
| Age 61–75 years | 2.293 | 1.400 | 3.752 | <0.001 |
| >75 years | 3.411 | 0.941 | 12.369 | 0.062 |
| HbA_1c_ (%) | 0.288 | 0.257 | 0.323 | <0.001 |
| Mean number of daily sensor scans | 1.014 | 1.010 | 1.018 | <0.001 |
| Coefficient of variation (%) | 0.886 | 0.874 | 0.898 | <0.001 |
| Co-payment bracket: < €18,000/year | 1.098 | 0.876 | 1.377 | 0.416 |
| Co-payment bracket: ≥ €18,000/year | 1.511 | 1.179 | 1.937 | <0.001 |

Goodness of fit (H. Lemeshow) 6.748, p<0.564; AUC: 0.835 [0.821; 0.848], p<0.001

***Table 3.6. Multivariable logistic regression model for achievement of glycemic control targets: TIR***

*Independent factors associated with achievement of the TIR glycemic target. Adjusted odds ratios (ORs) with 95% confidence intervals (95% CIs) and p values are reported. Model discrimination was assessed using the area under the receiver operating characteristic curve (AUC), and calibration was evaluated using the Hosmer–Lemeshow goodness-of-fit test. The co-payment categories “€18,000–€100,000/year” and “≥ €100,000/year” were combined due to the small sample size of the highest income bracket, in order to improve the robustness of the estimates.*

| Variable | OR | 95% CI lower limit | 95% CI upper limit | p value |
| --- | --- | --- | --- | --- |
| Male | 1.133 | 0.982 | 1.309 | 0.088 |
| Age 31–45 years | 1.273 | 1.020 | 1.588 | 0.033 |
| Age 46–60 years | 1.730 | 1.312 | 2.280 | <0.001 |
| Age 61–75 years | 2.157 | 1.386 | 3.360 | <0.001 |
| >75 years | 3.249 | 0.992 | 10.646 | 0.052 |
| HbA_1c_ (%) | 0.296 | 0.268 | 0.327 | <0.001 |
| Mean number of daily sensor scans | 1.015 | 1.010 | 1.019 | <0.001 |
| Coefficient of variation (%) | 0.968 | 0.958 | 0.978 | <0.001 |
| Co-payment bracket: < €18,000/year | 1.167 | 0.960 | 1.419 | 0.121 |
| Co-payment bracket: ≥ €18,000/year | 1.450 | 1.163 | 1.809 | <0.001 |

Goodness of fit (H. Lemeshow) 23.957, p<0.002; AUC: 0.794 [0.78; 0.81], p<0.001

***Table 3.7. Multivariable logistic regression model for achievement of glycemic control targets: TAR***

*Independent factors associated with achievement of the TAR glycemic target. Adjusted odds ratios (ORs) with 95% confidence intervals (95% CIs) and p values are reported. Model discrimination was assessed using the area under the receiver operating characteristic curve (AUC), and calibration was evaluated using the Hosmer–Lemeshow goodness-of-fit test. The co-payment categories “€18,000–€100,000/year” and “≥ €100,000/year” were combined due to the small sample size of the highest income bracket, in order to improve the robustness of the estimates.*

| Variable | OR | 95% CI lower limit | 95% CI upper limit | p value |
| --- | --- | --- | --- | --- |
| Male | 1.203 | 1.022 | 1.416 | 0.026 |
| Age 31–45 years | 0.878 | 0.689 | 1.118 | 0.291 |
| Age 46–60 years | 0.712 | 0.524 | 0.968 | 0.030 |
| Age 61–75 years | 1.007 | 0.564 | 1.797 | 0.982 |
| >75 years | 0.266 | 0.047 | 1.519 | 0.113 |
| HbA_1c_ (%) | 2.212 | 2.011 | 2.433 | <0.001 |
| Mean number of daily sensor scans | 0.995 | 0.991 | 0.999 | 0.018 |
| Coefficient of variation (%) | 0.783 | 0.770 | 0.797 | <0.001 |
| Co-payment bracket: < €18,000/year | 1.004 | 0.807 | 1.250 | 0.971 |
| Co-payment bracket: ≥ €18,000/year | 0.865 | 0.671 | 1.115 | 0.263 |

Goodness of fit (H. Lemeshow) 14.128 p<0.078; AUC: 0.851 [0.839; 0.863], p<0.001

***Table 3.8. Multivariable logistic regression model for achievement of glycemic control targets: TBR***

*Independent factors associated with achievement of the TBR glycemic target. Adjusted odds ratios (ORs) with 95% confidence intervals (95% CIs) and p values are reported. Model discrimination was assessed using the area under the receiver operating characteristic curve (AUC), and calibration was evaluated using the Hosmer–Lemeshow goodness-of-fit test. The co-payment categories “€18,000–€100,000/year” and “≥ €100,000/year” were combined due to the small sample size of the highest income bracket, in order to improve the robustness of the estimates.*
